# Facile one-step radio frequency magnetron sputtering of Ni/NiO on stainless steel for an efficient electrode for hydrogen evolution reaction

**DOI:** 10.3762/bjnano.16.63

**Published:** 2025-06-06

**Authors:** Ha Huu Do, Khac Binh Nguyen, Phuong N Nguyen, Hoai Phuong Pham

**Affiliations:** 1 NTT Hi-Tech Institute, Nguyen Tat Thanh University, Ho Chi Minh City 700000, Vietnam; 2 Institute of Applied Materials Science, Vietnam Academy of Science and Technology (VAST), 29TL Street, Ward Thanh Loc, District 12, Ho Chi Minh City 700000, Vietnam

**Keywords:** electrocatalysts, magnetron sputtering, nickel, nickel oxide, water electrolysis

## Abstract

The advancement of affordable, ultrastable, and efficient electrode materials for basic hydrogen evolution reaction (HER) plays a crucial role in industrial hydrogen manufacture, resolving problems caused by carbon dioxide emissions. Ni-based electrocatalysts have been well accepted as potential candidates to replace Pt-based electrocatalysts for HER because of their suitable Gibbs free hydrogen adsorption energy, good intrinsic catalytic properties, and high stability. However, solution-based synthetic approaches can be highly harmful to human beings. In this study, Ni/NiO nanolayers were prepared on stainless steel (SS) via a facile one-step radio frequency magnetron sputtering with various O_2_ flow rates. The O_2_ flow rate not only changed the crystal phase but also affected the morphology and atomic ratio of materials, leading to optimized HER efficiency. The evaluation of catalytic activities revealed that the optimal sample of Ni/NiO/SS-10 displayed a higher HER performance than bare SS. To produce H_2_ at a current density of 10 mA·cm^−2^, this electrode required a low overpotential of 184 mV and demonstrated remarkable durability over 12 h of operation. The high efficiency is attributed to the collaborative work of the NiO and Ni metal components and the good electrical conductivity of SS, which is advantageous for dissociative adsorption of water molecules, recombination of hydrogen atoms, and improvement of electronic/ionic motion. This work may introduce a facile and eco-friendly strategy for fabricating noble metal-free, efficient nanomaterials for electrocatalytic HER.

## Introduction

The world is facing a critical challenge through the increasing consumption of fossil fuels, causing CO_2_ emissions and resulting in climate change [1]. Hydropower can be harnessed to provide sustainable energy for future generations because there are no harmful emissions with only water vapor as a byproduct [2–4]. Also, hydropower offers better energy efficiency compared to gasoline, coal, and natural gas. Currently, hydrogen produced by water electrolysis is well accepted as an ecologically clean, sustainable method compared to other techniques, such as coal gasification and steam methane reforming [5–10]. According to reported works, Pt catalysts were recognized as the best material for electrochemical hydrogen evolution reaction (HER) [11–13]. However, it is challenging to use Pt-based nanomaterials for industrial applications because of their non-abundance and high cost. As a result, many studies have explored Pt-free catalysts, such as MoS_2_, WS_2_, CoSe_2_, and NiSe_2_, which displayed potential performance in acidic media [14–16]. However, their HER efficacy is poor in alkaline environments because of the lack of available protons, resulting in a high required voltage to cleave H–OH bonds. To resolve this problem, many groups fabricated metal/metal oxide-based nanomaterials using various solution-based methods for alkaline HER because of the efficacy of metal oxides in breaking water molecules. In this context, Ni/NiO-based nanomaterials were evaluated as promising catalysts for industrial applications because of their Gibbs free energy of hydrogen/water adsorption. Also, inexpensiveness and high durability are positive aspects regarding large-scale applications. For instance, Oshchepkov and coworkers revealed that the efficacy of NiO in cleaving H–OH bonds accelerated the formation of hydrogen on a Ni metal catalyst [17]. Yan and coworkers prepared Ni/NiO nanosheets via a hydrothermal process and annealing, which gave a high HER efficiency [18]. Wang et al. found that Ni metal plays a crucial role in NiO*_x_*-based material for water electrolysis [19]. However, using chemical methods to fabricate Ni/NiO-based nanomaterials is accompanied by toxic solvents and gases, influencing the environment and people’s health. One of the most significant current discussions in water electrolysis is finding green methods to prepare high-efficiency electrocatalysts. Thus, vacuum physical techniques have gained considerable attention in synthesizing electrode materials in recent years because they offer a cleaner pathway than solution-based synthetic processes [20–22]. Among vacuum deposition methods, magnetron sputtering has been widely applied in industrial applications for fabricating thin films because of its advantages, such as good adhesion and uniform distribution of materials on various substrates [23–25]. For instance, Ren et al. used the magnetron sputtering method to introduce Si into an iridium electrode to achieve efficient water electrolysis [26]. Additionally, this technique’s fast deposition rate and high level of the preserved substrate are definite advantages. Although studies have recognized the effectiveness of magnetron sputtering, research has yet to systematically investigate the effect of the O_2_ flow rate on the HER efficiency of Ni/NiO catalysts.

Commercial stainless steel (SS) costs less than other conductive substrates such as nickel foam (NF), carbon cloth, and fluorine-doped tin oxide [27–29]. Also, SS displays high electrical conductivity, outstanding chemical stability, and good mechanical properties. Therefore, numerous researchers have used SS as a template to deposit nanomaterials using various electrocatalytic applications [30]. For instance, Wang et al. deposited nickel–iron on SS, which was used as a high-performance electrode for water oxidation [31]. Hence, in this study, we utilized commercial 304 SS and coated it with the Ni/NiO catalyst through a one-step radio frequency (RF) magnetron sputtering route with various O_2_ flow rates, including 5, 10, 15, and 20 sccm. The electrode showed a higher HER efficacy than SS and Ni/SS, indicating the crucial role of NiO in water splitting. Moreover, the optimal sample Ni/NiO/SS-10 exhibited remarkable durability after 12 h of operation, suggesting great potential regarding industrial application.

## Results and Discussion

**Figure 1:**
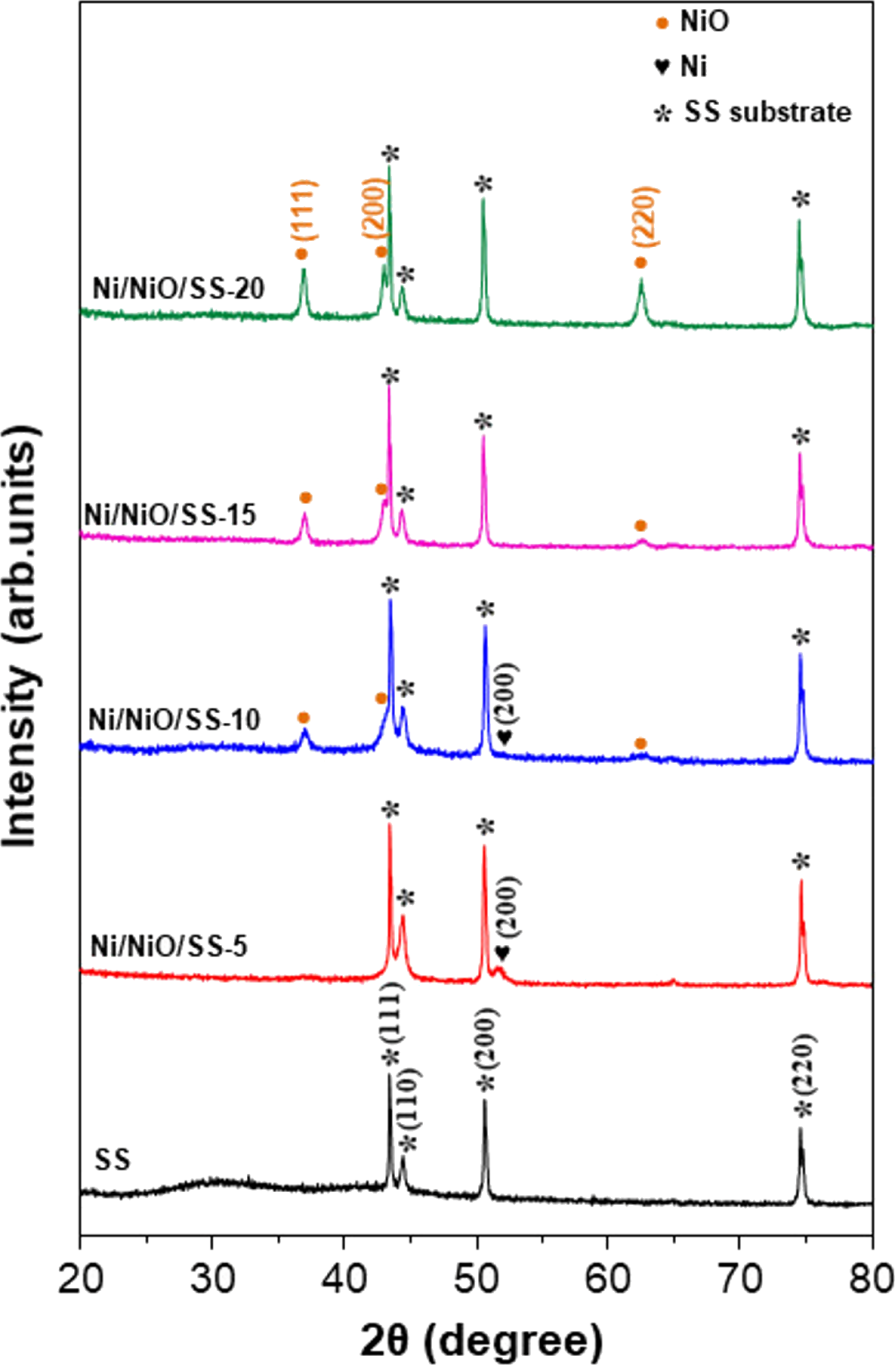
XRD patterns of SS, Ni/NiO/SS-5, Ni/NiO/SS-10, Ni/NiO/SS-15, and Ni/NiO/SS-20 electrodes.

Crystal structure and phase of the as-synthesized electrodes were verified by X-ray diffraction (XRD) measurements with 2θ ranging from 20° to 80°. Figure 1 displays the XRD patterns of SS, Ni/NiO/SS-5, Ni/NiO/SS-10, Ni/NiO/SS-15, and Ni/NiO/SS-20 electrodes. Regarding the commercial 304 SS sample, the typical peaks at 43.64°, 50.68°, and 74.56° were indexed to the (111), (200), and (220) crystal planes of the cubic γ-Ni–Cr–Fe phase [27–33]. At a low O_2_ flow rate (5 sccm), a Ni metal phase appeared, visible by the peak at 51.9°, identical to the Ni/SS sample (Supporting Information File 1, Figure S1), whereas the NiO phase also began to appear with low-intensity peaks. When the O_2_ flow rate was increased from 10 to 20 sccm in the synthetic process, the peak intensity of the NiO phase was enhanced. More importantly, the XRD image of Ni/NiO/SS-10 presents peaks at 37.20°, 43.21°, and 62.91°, which correspond to the (111), (200), and (220) planes of the NiO phase (PDF 00-004-0835) [34–35]. Meanwhile, the peak of the Ni metal phase still appeared in the Ni/NiO/SS-10 sample, proving the co-existence of metal and metal oxide phases, offering a potential for HER. These outcomes indicated the successful Ni/NiO thin film fabrication on SS substrates. The uniformity of the electrocatalyst material is a vital factor that has a direct effect on electrode performance.

**Figure 2:**
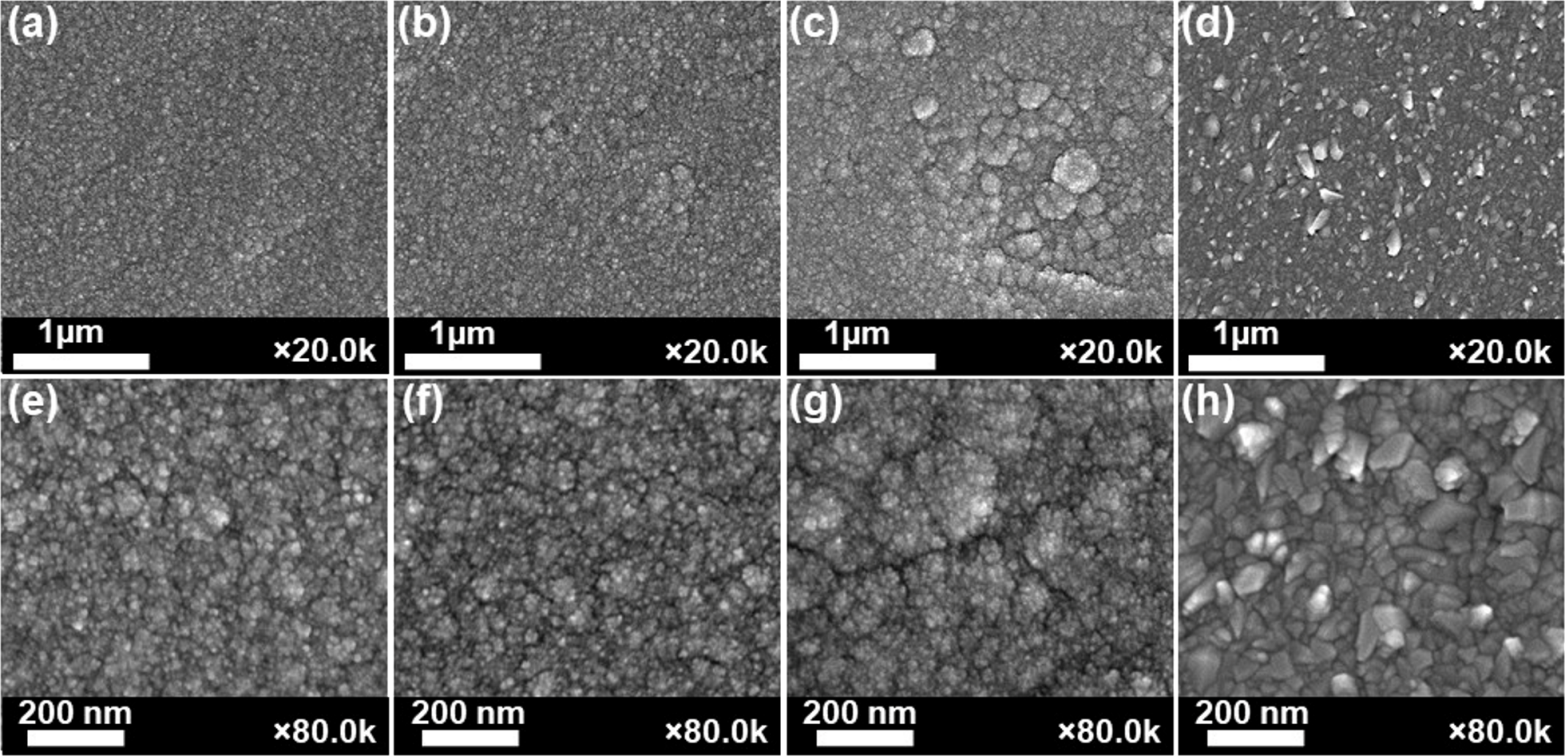
SEM images of (a, e) Ni/NiO/SS-5, (b, f) Ni/NiO/SS-10, (c, g) Ni/NiO/SS-15, and (d, h) Ni/NiO/SS-20 electrodes.

Scanning electron microscopy (SEM) was carried out to analyze the morphology of the Ni/NiO thin film on the SS substrate. The as-synthesized Ni/NiO film at 5 sccm of O_2_ flow rate showed uniform and continuous appearance on the entire surface of the SS substrate (Figure 2a,e). However, at high O_2_ flow rates, the surface of Ni/NiO/SS-10, Ni/NiO/SS-15, and Ni/NiO/SS-20 electrodes became rougher, as shown in Figure 2b–d, which was attributed to a higher metal oxide content. More importantly, the composition of the samples (wt %) changed with different O_2_ flow rates in the sputtering process. The proportion of O increases when the O_2_ flow rate is increased, determined by energy-dispersive X-ray spectroscopy (EDX), as shown in Table 1. Ni/NiO/SS-5 displayed the lowest O content (4.69 wt %). In contrast, Ni/NiO/SS-20 showed the highest O content (22.69 wt %), attributed to the highest O_2_ flow rate in the sputtering process. Ni/NiO/SS-10 exhibited a moderate O_2_ content (11.96 wt %), which could bring the highest HER efficiency. The Ni/NiO ratio is the most crucial parameter in the Ni/NiO catalyst system, influencing the electrode’s HER efficiency, which Yan and coworkers proved [18].

**Table 1:** Nickel and oxygen fractions from EDX analyses.

Electrodes	Ni (wt %)	O (wt %)

Ni/NiO/SS-5	95.31	4.69
Ni/NiO/SS-10	88.04	11.96
Ni/NiO/SS-15	82.93	17.07
Ni/NiO/SS-20	77.31	22.69

**Figure 3:**
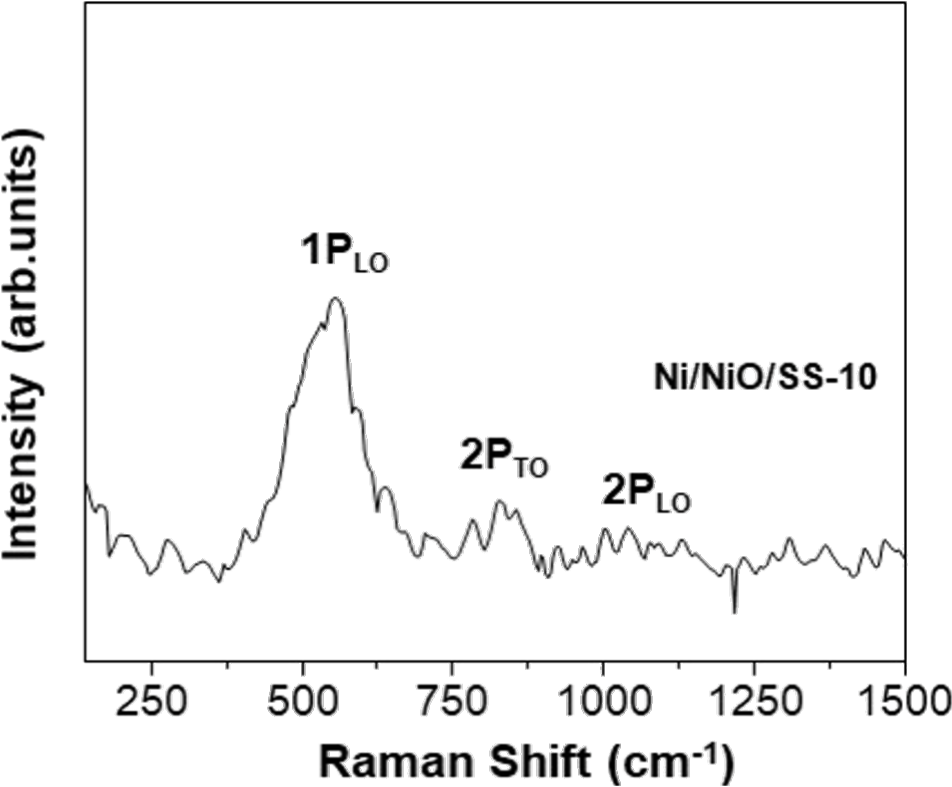
Raman spectrum of the Ni/NiO/SS-10 electrode.

**Figure 4:**
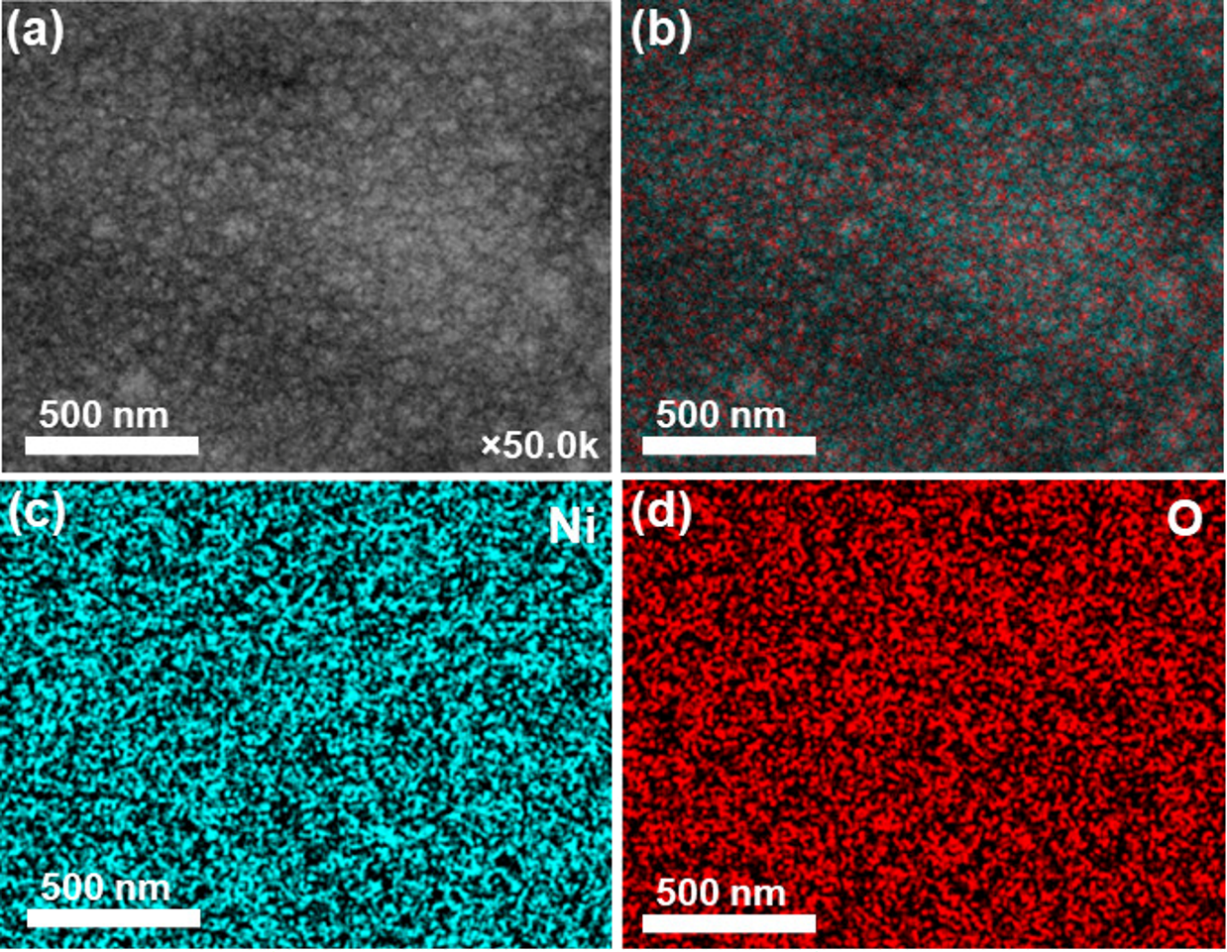
(a) SEM image, (b) overall element mapping, and (c) nickel and (d) oxygen element mapping of the Ni/NiO/SS-10 sample.

**Figure 5:**
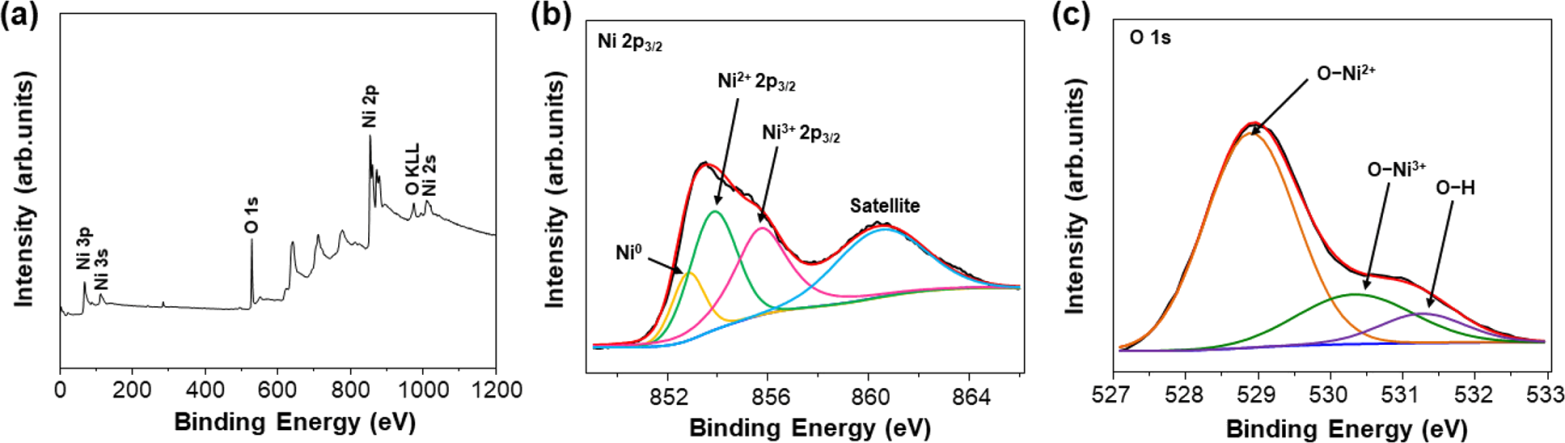
(a) Survey XPS spectrum of Ni/NiO/SS-10. High-resolution XPS spectra of (b) Ni 2p_3/2_ and (c) O 1s.

The Raman spectrum of the Ni/NiO/SS-10 electrode displayed the prominent peaks shown in Figure 3. The bands at 200 to 600 cm^−1^ represent one phonon (1P), whereas the bands at 650 to 1100 cm^−1^ could be assigned to two phonons (2P) of NiO species in the electrode. In particular, the Raman peak at 552 cm^−1^ was indexed into the 1P longitudinal optical (LO) mode, whereas the peak at 1052 cm^−1^ is attributed to the 2P_LO_ mode of the Ni–O bonds. These peaks indicate the existence of Ni defects in the Ni/NiO/SS-10 sample, which is favorable for electrocatalytic applications [36–38]. Additionally, Figure 4 depicts EDX analysis and proves the uniform distribution of the primary elements (Ni, O) in the Ni/NiO/SS-10 sample. This outcome revealed that catalytic sites were also uniformly distributed on the electrode’s surface. Figure 5a exhibits the X-ray photoelectron spectroscopy (XPS) survey of the Ni/NiO/SS-10 sample. It can be seen that Ni/NiO/SS gives Ni and O peaks. The chemical state of Ni is further analyzed by the Ni 2p_3/2_ peaks. As depicted in Figure 5b, the Ni 2p_3/2_ spectrum can be deconvoluted into four peaks. The peaks at 852.8, 853.7, and 855.8 eV correspond to Ni^0^, Ni^2+^, and Ni^3+^, respectively. Another broad peak at nearly 861 eV is attributed to a satellite peak. The presence of Ni^3+^ can be ascribed to the formation of NiOOH species originating from water adsorption on the surface of NiO. The high-resolution O 1s spectrum can be deconvoluted into three peaks, namely, O–Ni^2+^ (528.9 eV), O–Ni^3+^ (530.5 eV), and O–H (531.4 eV) [39].

**Figure 6:**
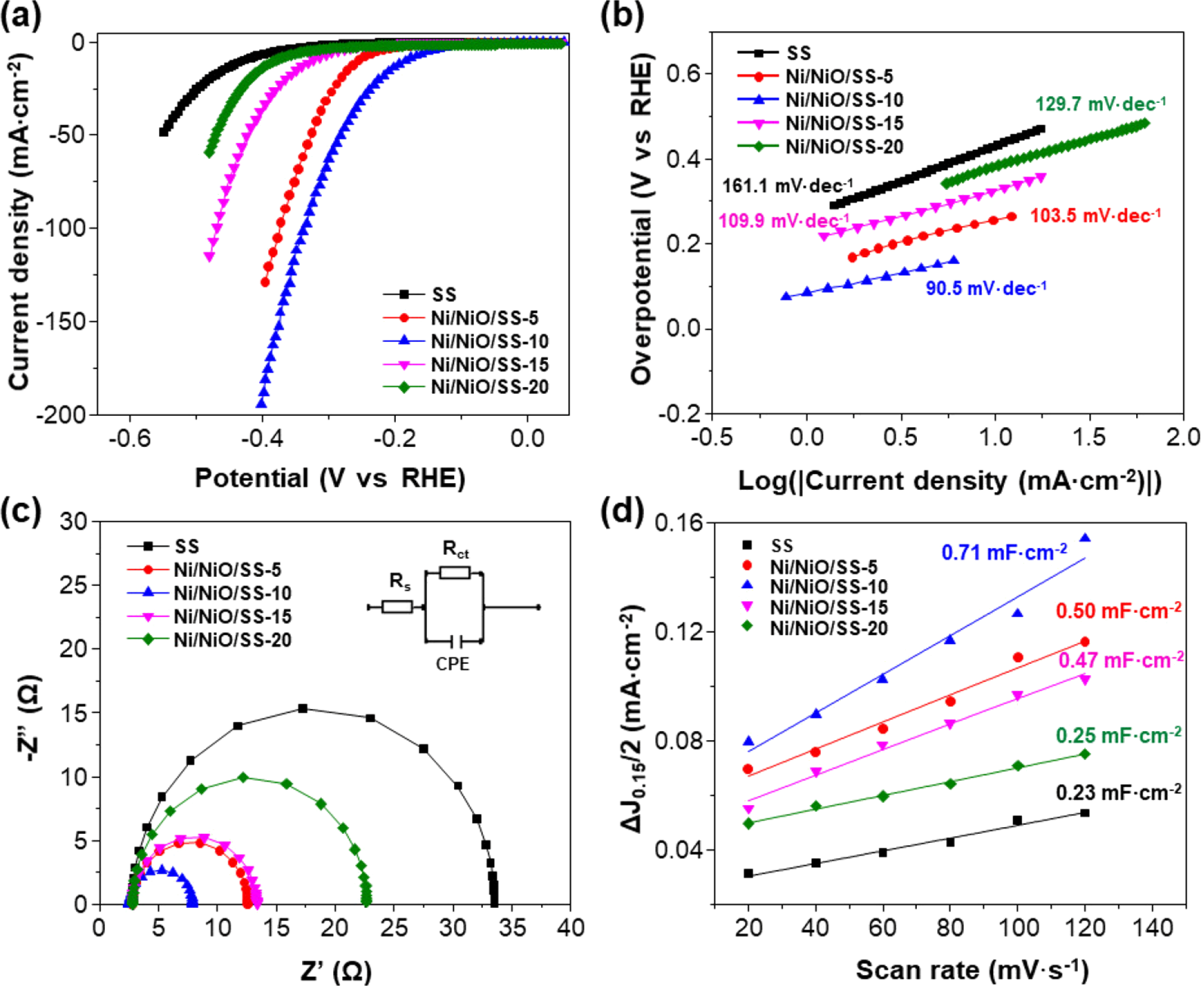
(a) Polarization curves of SS, Ni/NiO/SS-5, Ni/NiO/SS-10, Ni/NiO/SS-15, and Ni/NiO/SS-20 electrodes. (b) Corresponding Tafel slopes of electrodes. (c) Nyquist plots of different samples recorded at a potential of −200 mV vs RHE. (d) Double-layer capacitances obtained using cyclic voltammetry (CV).

To find the optimal O_2_ flow rate for HER applications, we evaluated the HER catalytic activities of the SS, Ni/NiO/SS-5, Ni/NiO/SS-10, Ni/NiO/SS-15, and Ni/NiO/SS-20 electrodes. Linear sweep voltammetry (LSV) of electrodes was studied in a solution of 1.0 M KOH. As shown in Figure 6a, Ni/NiO/SS-10 provides better HER efficacy than other as-synthesized catalysts. To reach a cathodic current density of 10 mA·cm^−2^, SS, Ni/NiO/SS-5, Ni/NiO/SS-10 Ni/NiO/SS-15, and Ni/NiO/SS-20 electrodes needed overpotentials of 431, 247, 184, 326, and 382 mV vs RHE, respectively. A smaller overpotential implies a smaller energy barrier for water electrolysis to produce hydrogen. These outcomes indicate that Ni/NiO/SS-10 shows the highest HER efficiency among the investigated electrodes. Its overpotential is comparable with Ni-based nanomaterials in the literature, as depicted in Table 2 [40–48]. Specifically, the overvoltage of Ni/NiO/SS-10 is lower than those of NiO/C (565 mV) [40], Ni_3_S_2_/NC (199 mV) [44], NiCoP/rGO (209 mV) [47], and NF-Ni_3_Se_2_/Ni (203 mV) [48]. Also, the Tafel slopes indicate that the reaction kinetics of the Ni/NiO/SS-10 electrode is faster than those of the other electrodes (Figure 6b). Notably, the Tafel slopes are 161.1, 103.5, 90.5, 109.9, and 129.7 mV·dec^−1^ for SS, Ni/NiO/SS-5, Ni/NiO/SS-10, Ni/NiO/SS-15, and Ni/NiO/SS-20 electrodes, respectively. Moreover, the Tafel slope value of Ni/NiO/SS-10 is lower than the published ones of Ni/NiO (114 mV·dec^−1^) [41], NiP_2_/NiO (94 mV·dec^−1^) [45], Ni_5_P_4_ (98 mV·dec^−1^) [46], and NiCoP/rGO (124.1 mV·dec^−1^) [47]. Generally, Tafel slopes are used to determine the HER mechanism, which could follow the Volmer–Heyrovsky or Volmer–Tafel pathway [49]. In this work, the Tafel value of Ni/NiO/SS-10 is between 40 and 120 mV·dec^−1^, revealing that the HER mechanism follows the Volmer–Heyrovsky mechanism.

**Table 2:** Summary of alkaline HER performance for various Ni-based electrocatalysts.

Catalysts	Current density (mA·cm^−2^)	Overpotential (mV)	Tafel slope (mV·dec^−1^)	Reference

NiO/C	10	565	77.8	[40]
Ni/NiO	10	120	114	[41]
N-NiO	10	154	90	[42]
Ni_3_S_2_@2DCo-MOF	10	140	90.3	[43]
Ni_3_S_2_/NC	10	199	72	[44]
NiP_2_/NiO	10	≈131	94	[45]
Ni_5_P_4_	10	49	98	[46]
NiCoP/rGO	10	209	124.1	[47]
NF-Ni_3_Se_2_/Ni	10	203	79	[48]
NiO/SnO_2_	10	157	155	[50]
NiO@TiO_2_	10	144	152.34	[51]
Ni/NiO/SS-10	10	184	90.5	this work

During the electrocatalytic process, the NiO components are crucial in facilitating the dissociative adsorption of water molecules to generate adsorbed H atoms (H_ads_). Also, NiO units are vital in scavenging OH^−^ ions, which could reduce the catalytic activity of metallic Ni phases [52]. Meanwhile, metallic Ni phases favor the recombination of H_ads_ to produce H_2_ gas, implying a perfect pair of Ni/NiO for the HER catalytic process [53–55]. Therefore, the Ni/NiO/SS-10 electrode exhibits higher HER activity than SS and Ni/SS, as shown in Supporting Information File 1, Figure S2. More importantly, the highest HER efficiency of the Ni/NiO/SS-10 electrode could be explained as follows: At a low O_2_ flow rate (5 sccm), NiO is formed with low content, resulting in a decrease in the dissociative adsorption of water molecules. In contrast, at high O_2_ flow rates (15 and 20 sccm), NiO is produced with a large ratio of NiO/Ni, which is indicated by XRD peaks of NiO with high intensity. A high NiO concentration prefers the dissociative adsorption of water molecules, while hydrogen desorption is limited. Besides, the O content increased in the order of Ni/NiO/SS-5 < Ni/NiO/SS-10 < Ni/NiO/SS-15 < Ni/NiO/SS-20, confirmed by EDX analysis. Overall, the material obtained at 10 sccm O_2_ flow rate has the most suitable O/Ni ratio among the four materials and thus exhibited the best HER efficiency [18].

Electrochemical impedance spectroscopy (EIS) was carried out at a voltage of −200 mV to confirm the HER kinetics. Figure 6c shows the Nyquist plots of the various electrodes accompanied by an equivalent circuit (inset of Figure 6c). Ni/NiO/SS-10 has a charge transfer resistance (*R*_ct_) of 5.35 Ω, which is much smaller than that of SS (30.75 Ω), Ni/NiO/SS-5 (9.79 Ω), Ni/NiO/SS-15 (10.58 Ω), and Ni/NiO/SS-20 (19.87 Ω). The lowest *R*_ct_ of Ni/NiO/SS-10 indicates the best electron/ion transfer kinetics for HER, consistent with the Tafel slope analysis. In general, the electrochemical surface area (ECSA) can be predicted by measuring the double-layer capacitance (*C*_dl_), which is derived from the CV technique at various scan rates, ranging from 20 to 120 mV·s^−1^, as depicted in Suporting Information File 1, Figure S4. Notably, the *C*_dl_ of Ni/NiO/SS-10 is 0.71 mF·cm^−2^, which is higher than those of Ni/NiO/SS-5 (0.50 mF·cm^−2^), Ni/NiO/SS-15 (0.47 mF·cm^−2^), Ni/NiO/SS-20 (0.25 mF·cm^−2^), and SS (0.23 mF·cm^−2^), offering its higher ECSA along with a remarkable HER efficacy (Figure 6d).

**Figure 7:**
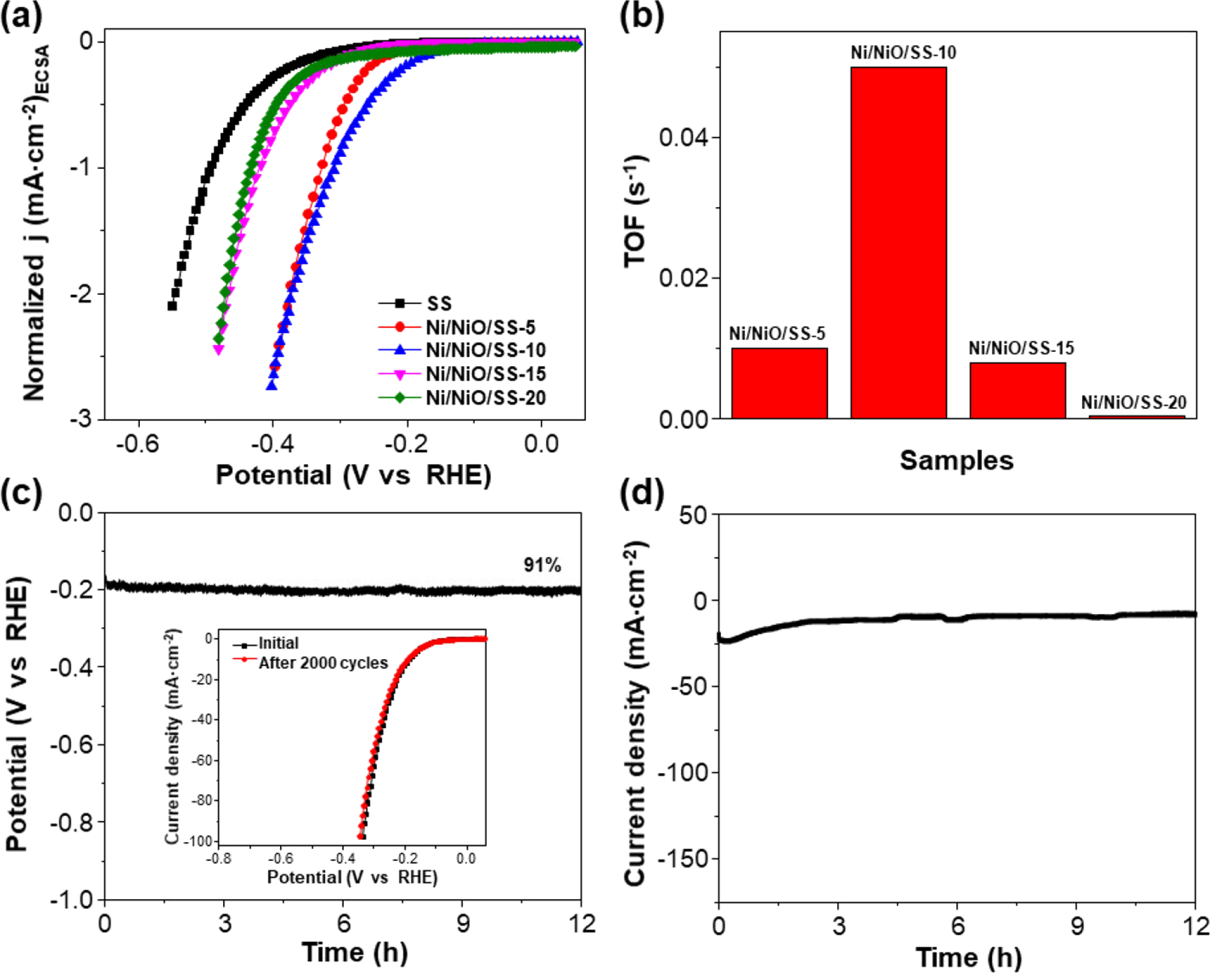
(a) LSV curves (normalized to the ECSA) of SS, Ni/NiO/SS-5, Ni/NiO/SS-10, Ni/NiO/SS-15, and Ni/NiO/SS-20 electrodes. (b) TOFs of Ni/NiO/SS-5, Ni/NiO/SS-10, Ni/NiO/SS-15, and Ni/NiO/SS-20 electrodes at an overpotential of 200 mV. (c) Potential–time response of the Ni/NiO/SS-10 electrode measured over a period of 12 h. Inset: Polarization curves recorded initially and after 2000 CV cycles of the Ni/NiO/SS-10 electrode. (d) Chronoamperometric curve of Ni/NiO/SS-10 electrode recorded for 12 h.

To assess the intrinsic catalytic properties, the LSV curves were normalized to the ECSA, as displayed in Figure 7a. Ni/NiO/SS-10 presents a better intrinsic HER catalytic activity than the other samples. Also, the turnover frequency (TOF) is a vital factor for investigating the intrinsic catalytic activities of electrodes for the HER [56]. Hence, we determined the TOF of different electrodes at an overpotential of 200 mV for comparison. The TOF of Ni/NiO/SS-10 is 0.051 s^−1^, which is higher than those of Ni/NiO/SS-5 (0.010 s^−1^), Ni/NiO/SS-15 (0.008 s^−1^), and Ni/NiO/SS-20 (0.004 s^−1^), as shown in Figure 7b. This outcome implies that Ni/NiO/SS-10 exhibits a better efficiency regarding the active sites in the electrocatalytic HER process. Finally, we evaluated the long-term electrochemical stability of the Ni/NiO/SS-10 electrode via three methods, that is, chronopotentiometry, CV, and chronoamperometry. The potential–time response revealed that approximately 91% of the initial voltage is retained after 12 h of continuous hydrogen production at a cathodic current density of 10 mA·cm^−2^ (Figure 7c). Also, the LSV curve of Ni/NiO/SS-10 exhibits minimal changes after 2000 cycles (inset of Figure 7c). Additionally, the current density was maintained during the 12 h testing of chronoamperometry, as depicted in Figure 7d. This indicates the high stability of the Ni/NiO/SS-10 electrode for alkaline water electrolysis. To explain the high HER efficiency of the Ni/NiO/SS-10 electrode, we analyzed SEM and EDX after the stability test (Supporting Information File 1, Figure S5). It is noted that there is no significant change in the morphological structure and elemental distribution of Ni/NiO/SS-10. However, EDX exhibits a slight increase in the weight fraction of oxygen. A possible explanation for this might be that OH^−^ ions are adsorbed by NiO species [57]. Another possible explanation is that air oxidizes the electrode after chronoamperometry [36].

## Conclusion

Ni/NiO/SS self-standing electrodes were successfully prepared through a facile magnetron sputtering technique and were used as high-efficiency cathodes for the HER. By controlling the O_2_ flow rate of 10 sccm in the reactor, the HER performance of the Ni/NiO/SS-10 electrode was optimized to a low overpotential of 184 mV at a current density of 10 mA·cm^−2^ and a moderate Tafel slope of 90.5 mV·dec^−1^. Also, remarkable stability was recorded after 12 h continuous hydrogen production by chronopotentiometry and chronoamperometry, and 2000 cycles of CV. This performance was ascribed to the collaborative work of the NiO phases, metallic Ni, and high conductivity of SS, which is advantageous for the Volmer step in alkaline solution, the combination of adsorbed H atoms, and electron transport in the catalytic process. These conclusions guide the fabrication of binder-free, robust, affordable electrocatalysts using magnetron sputtering for basic HER.

## Experimental

### Chemicals and materials

Nickel target (99.95%), Ar gas (99.9995%), and O_2_ gas (99.9995%) were provided by Nippon Sanso company. KOH (99.95%) was provided by Sigma-Aldrich. Deionized water (DI) was created on a Millipore Milli-Q apparatus. Commercial 304 SS with a thickness of 1 mm was used as a substrate for catalyst growth.

### Fabrication of the Ni/NiO/SS samples

Grade 304 SS was cut into pieces of 60 mm × 25 mm, washed with soap, and then sonicated in a mixture of acetone and ethanol to remove the impurities left on the SS template. After that, thin films of Ni/NiO were deposited on the SS substrate through reactive RF magnetron sputtering with various O_2_ flow rates. In particular, the Ni/NiO nanolayers were deposited using a pure Ni target at a deposition pressure of 5 × 10^−3^ Torr, sputtering power of 70 W, and substrate temperature of 250 °C. In this process, the Ar flow rate was kept at 80 sccm, whereas the O_2_ flow rates were to 0, 5, 10, 15, and 20 sccm to create various electrodes, designated Ni/SS, Ni/NiO/SS-5, Ni/NiO/SS-10 Ni/NiO/SS-15, and Ni/NiO/SS-20, respectively. The obtained products were stored under vacuum for further analysis.

### Materials characterization

The crystal structure of materials was confirmed by XRD using Cu Kα radiation with a wavelength of 0.154 nm on a X-ray diffractometer (D8 Advance, Bruker). The morphology of the obtained products was analyzed utilizing SEM on an S-4800 Hitachi. Chemical components and element distribution in materials were studied using EDX. The Raman spectra were studied using a LabRAM-HR Evolution Raman microscope with a laser wavelength of 532 nm. The composition of the thin films was investigated using XPS on a Thermo Scientific K-Alpha XPS system.

### Electrochemical measurements

The HER catalytic activities were assessed on an electrochemical workstation (VMP-3e Multichannel Potentiostat, Biologic) in a three-electrode system. A Hg/HgO electrode was used as a reference electrode, while a carbon rod was utilized as the auxiliary electrode. The self-standing Ni/NiO on the SS substrate as a working electrode was cut into pieces of 1 cm × 0.5 cm and a catalyst loading area of 0.5 cm × 0.5 cm. LSV was carried out at a scan rate of 2 mV·s^−1^, followed by 85% *iR* compensation, in a solution of 1.0 M KOH. EIS was investigated at a potential of −200 mV vs a reversible hydrogen electrode (RHE) in a frequency zone from 10^5^ to 0.1 Hz. *C*_dl_ values of the samples were obtained by using CV in the non-faradaic potential range at different scan rates. The Nernst equation was used to convert the measured potential relative to Hg/HgO into RHE potential: *E*_RHE_ = *E*_Hg/HgO_ + 0.098 + 0.059pH.

## Supporting Information

File 1 XRD and HER performance of Ni/SS; EDX spectra of various Ni/NiO/SS electrodes; CV curves in non-faradaic zone of electrodes at various scan rates (20–120 mV·s^−1^) in 1.0 M KOH.

## Data Availability

Data generated and analyzed during this study is available from the corresponding author upon reasonable request.
