# Supplementary material for: Facile one-step radio frequency magnetron sputtering of Ni/NiO on stainless steel for an efficient electrode for hydrogen evolution reaction
Source: Beilstein J Nanotechnol. 2025 Jun 6;16:837–46. doi: 10.3762/bjnano.16.63 (PMC12152313; doi:10.3762/bjnano.16.63)
Supplement: File 1 — XRD and HER performance of Ni/SS; EDX spectra of various Ni/NiO/SS electrodes; CV curves in non-faradaic zone of electrodes at various scan rates (20–120 mV·s−1) in 1.0 M KOH. [file Beilstein_J_Nanotechnol-16-837-s001.pdf]

BEILSTEIN JOURNAL OF NANOTECHNOLOGY

Supporting Information

for

Facile one-step radio frequency magnetron sputtering of Ni/NiO on stainless steel for an efficient electrode for hydrogen evolution reaction

Ha Huu Do, Khac Binh Nguyen, Phuong N. Nguyen and Hoai Phuong Pham

Beilstein J. Nanotechnol. **2025,** *16,* 837–846. doi:10.3762/bjnano.16.63

XRD and HER performance of Ni/SS; EDX spectra of various Ni/NiO/SS electrodes; CV curves in non-faradaic zone of electrodes at various scan rates (20–120 mV·s^{-1}) in 1.0 M KOH

License and Terms: This is a supporting information file under the terms of the Creative Commons Attribution License (https://creativecommons.org/licenses/by/4.0). Please note that the reuse, redistribution and reproduction in particular requires that the author(s) and source are credited and that individual graphics may be subject to special legal provisions.
The license is subject to the *Beilstein Journal of Nanotechnology* terms and conditions: (https://www.beilstein-journals.org/bjnano/terms)

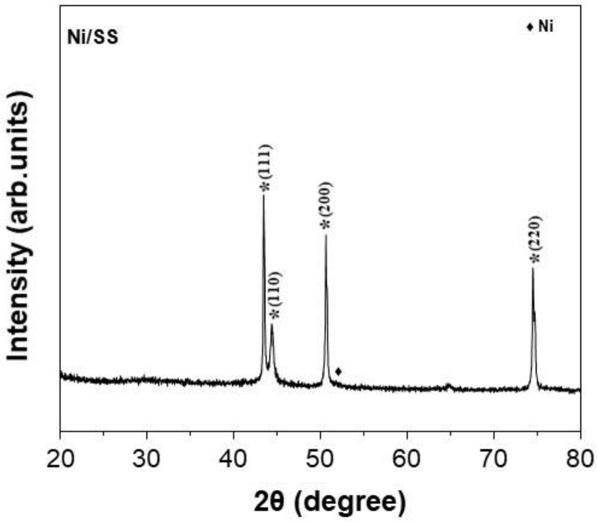

Figure S1: XRD data of Ni/SS.

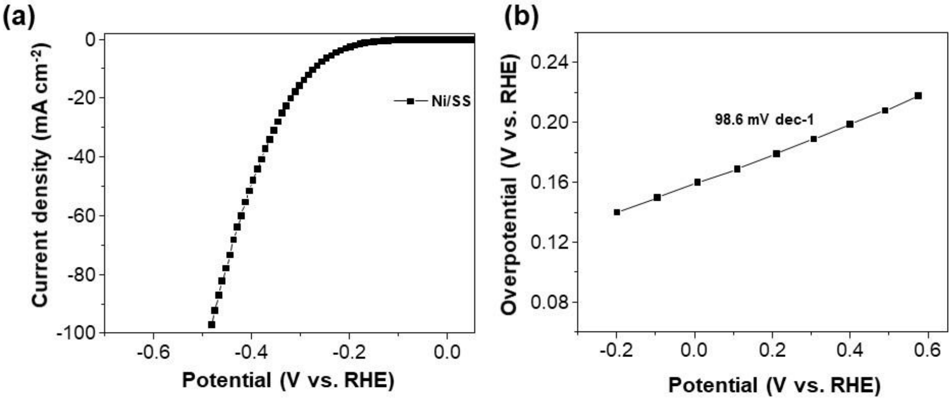

Figure S2: (a) Polarization curve of Ni/SS electrode, (b) corresponding Tafel slope of Ni/SS electrode.

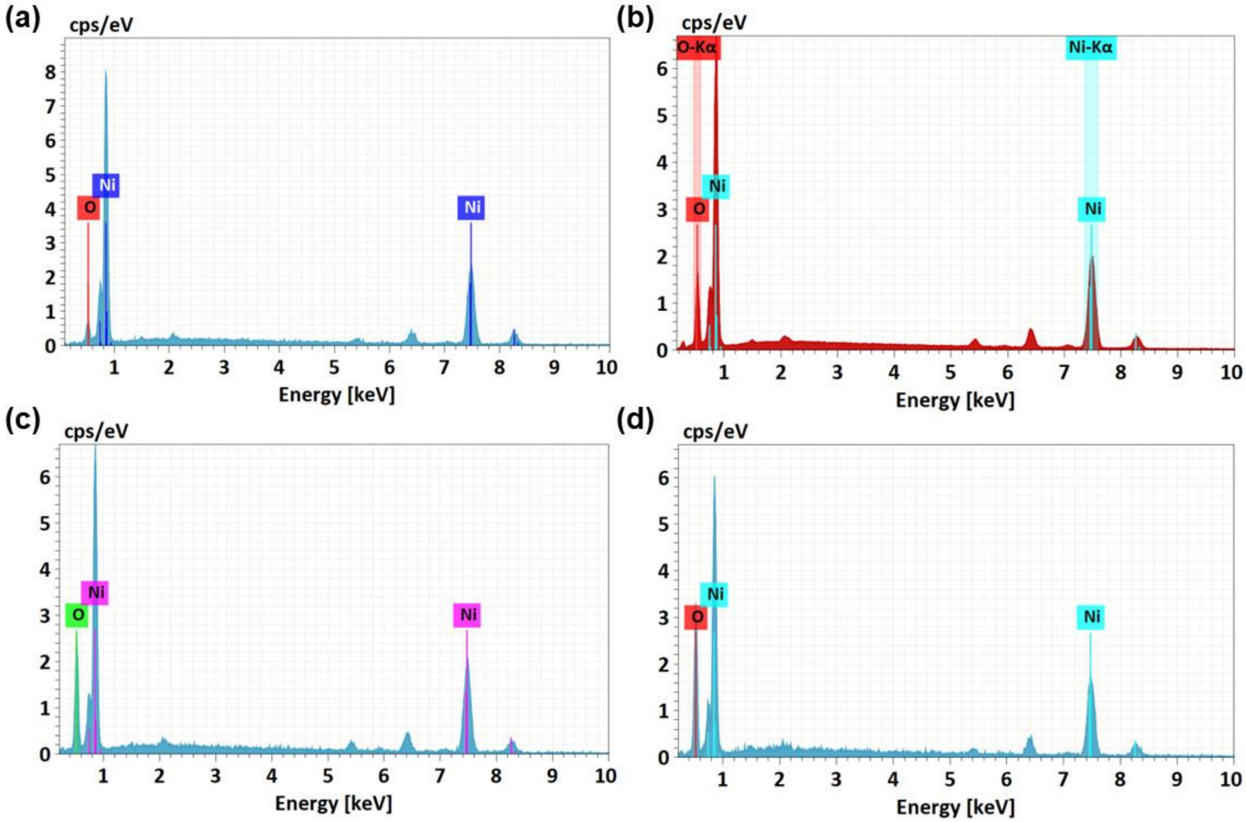

Figure S3: EDX spectra of (a) Ni/NiO/SS-5, (b) Ni/NiO/SS-10, (c) Ni/NiO/SS-15, (d) Ni/NiO/SS-20.

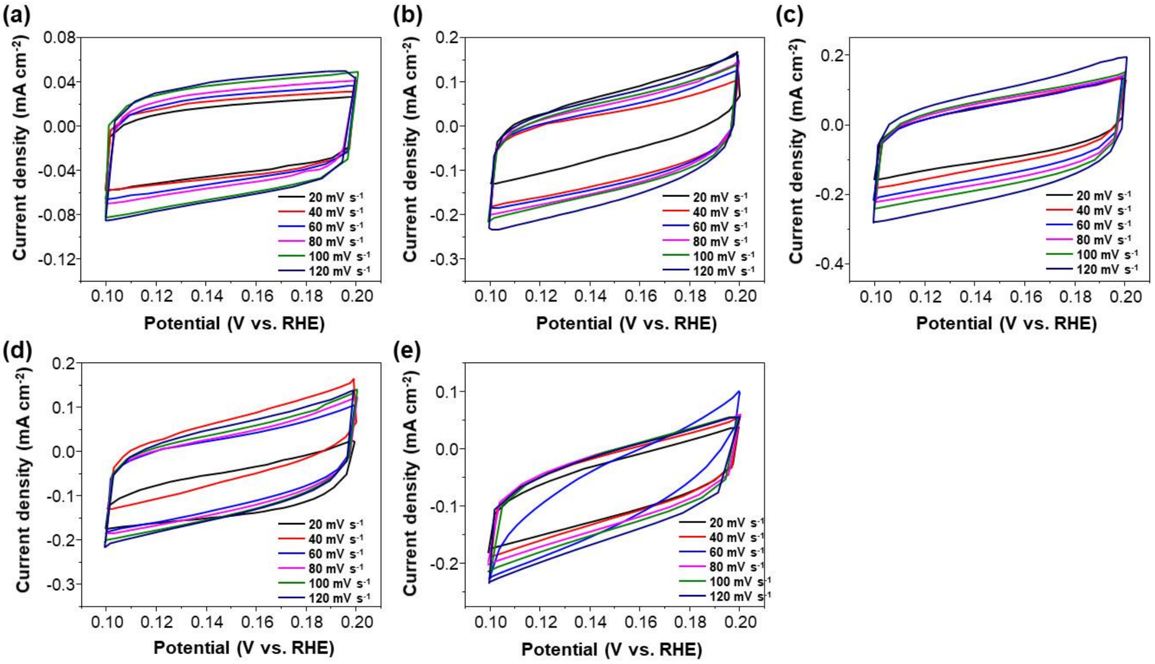

Figure S4: CV curves in non-faradaic zone of SS, Ni/NiO/SS-5, Ni/NiO/SS-10, Ni/NiO/SS-15, Ni/NiO/SS-20 at various scan rates (20–120 mV/s) in 1.0 M KOH.

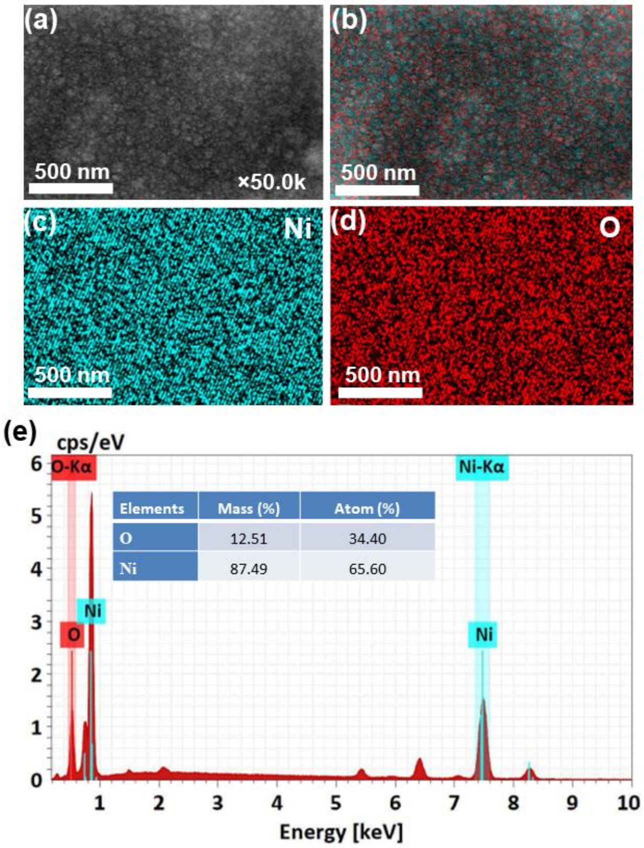

Figure S5: (a) SEM image, (b) Overall mapping elements corresponding to (c) nickel, (d) oxygen, (e) EDX analysis of Ni/NiO/SS-10 electrode after stability test.

Calculation of TOF

The number (N_{Ni}) of surface-active sites per cm^2 is calculated based on Eqs (S1) and (S2):

$$N_{Ni} = n_{Ni} \times N_A \quad \text{(S1)}$$

$$N_{Ni} = \frac{m(film)\, mg \times Wt.\%Ni}{58.693\times10^{3} mgmol^{-1}} \times 6.022 \times 10^{23} \frac{1}{mol} \quad \text{(S2)}$$

Wt.% is the weight percent of Ni in the sample.

The number of total hydrogen is calculated from the current density using Eqs (S3)

$$\left(j\,\frac{mA}{cm^2}\right)\left(\frac{1\,A}{1000\,mA}\right)\left(\frac{1\,{}^{C}/_{s}}{1\,A}\right)\left(\frac{1\,mol\,e^-}{96485.3\,C}\right)\left(\frac{1\,mol\,H_2}{2\,mol\,e^-}\right) \times$$

$$\left(\frac{6.022\times10^{23} molecules\,H_2}{1\,mol\,H_2}\right) = 3.12 \times 10^{15} \frac{{}^{H_2}/_{s}}{cm^2} per \frac{mA}{cm^2} \quad \text{(S3)}$$

Here j: ($mA\cdot cm^{-2}$) is the current density at an overpotential of 200 mV in 1.0 M KOH; therefore, the TOF per site is calculated as Eqs (S4).

$$\frac{\left(3.12\times10^{15}\frac{{}^{H_2}/_{s}}{cm^2}per\frac{mA}{cm^2}\right)\left(j\frac{mA}{cm^2}\right)}{(N_{Ni})} = \left(3.12 \times 10^{15} \times \frac{j}{N_{Ni}}\right)\frac{{}^{H_2}/_{s}}{surface} \quad \text{(S4)}$$
